# Store-Operated Calcium Entry and Its Implications in Cancer Stem Cells

**DOI:** 10.3390/cells11081332

**Published:** 2022-04-13

**Authors:** Isaac Jardin, Jose J. Lopez, Jose Sanchez-Collado, Luis J. Gomez, Gines M. Salido, Juan A. Rosado

**Affiliations:** Cellular Physiology Research Group, Department of Physiology, Institute of Molecular Pathology Biomarkers, University of Extremadura, 10003 Caceres, Spain; jjlopez@unex.es (J.J.L.); josesc@unex.es (J.S.-C.); luih@unex.es (L.J.G.); gsalido@unex.es (G.M.S.)

**Keywords:** store-operated calcium entry, Orai1, cancer stem cells

## Abstract

Tumors are composed by a heterogeneous population of cells. Among them, a sub-population of cells, termed cancer stem cells, exhibit stemness features, such as self-renewal capabilities, disposition to differentiate to a more proliferative state, and chemotherapy resistance, processes that are all mediated by Ca^2+^. Ca^2+^ homeostasis is vital for several physiological processes, and alterations in the patterns of expressions of the proteins and molecules that modulate it have recently become a cancer hallmark. Store-operated Ca^2+^ entry is a major mechanism for Ca^2+^ entry from the extracellular medium in non-excitable cells that leads to increases in the cytosolic Ca^2+^ concentration required for several processes, including cancer stem cell properties. Here, we focus on the participation of STIM, Orai, and TRPC proteins, the store-operated Ca^2+^ entry key components, in cancer stem cell biology and tumorigenesis.

## 1. Introduction

Normal stem cells are undifferentiated or partially differentiated cells that are characterized by their ability to self-renew, the process of bringing about indefinitely more cells of the same type, as well as to differentiate in more specialized mature cells. The term “stem cell” was coined by Ernst Haeckel in 1868 to describe the ancestor unicellular organism from which all multicellular organisms were supposed to evolve [1]. Normal stem cells can be found from the early embryos to the mature subject, where they can be present in different tissues, including the bone marrow, skin and hair follicles, muscle, brain, and epithelia, among others [2].

Cancer stem cells (CSC), also known as tumor-initiating cells, share features of both cancer and stem cells. These cells constitute a sub-population of tumor-resident malignant cells responsible for recurrence, metastasis formation, and chemoresistance. Experimental evidence indicates that CSC exhibit “stemness” properties, that is, the ability of cells to perpetuate their lineage, to bring about differentiated cells and to interact with their microenvironment to maintain a balance between quiescence, proliferation, and regeneration [3]. According to this, CSC exhibit low proliferative rates, self-renewing capacity, propensity to differentiate into proliferating tumor cells, resistance to apoptosis and senescence, as well as to chemo- and radio-therapy, evasion of immune attack, and are responsible for invasion and metastases [4,5].

CSC have been reported to derive from “normal” tissue resident stem cells or from differentiated cells undergoing transformation [6]. Furthermore, during cancer evolution, secondary self-renewing cell populations might arise, which supports the notion that the CSC phenotype might not be exclusively defined by the intrinsic characteristics of a cell but might also be determined by other phenomena, such as the microenvironment interaction [7]. The existence of CSC has been demonstrated in a variety of tumors, from leukemia [8] to solid tumors, such as colon [9], breast [10], brain [11], pancreatic [12], oral [13], esophageal [14], and liver [15] cancers as well as melanoma [16], among others. The functional role of CSC in tumor initiation is complex and not completely resolved. In contrast to the stochastic model that hypothesizes that most tumor cells can act as tumor initiating cells [17], the hierarchical or CSC hypothesis assumes that only a sub-population of cells, the CSC, have the ability for tumor initiation [18]. According to this hypothesis, CSC are responsible for the initiation, metastasis, chemotherapy resistance, and recurrence of the tumor [19]. Nevertheless, the CSC hypothesis applies to a limited number of cases. For instance, in certain tumors, such as testicular cancer, CSC are more sensitive to cisplatin than the differentiated tumor cells. Furthermore, in glioblastoma multiforme, a large number of differentiated tumor cells survive after anti-tumoral therapy, not just a small sub-population of CSC, and many of the surviving cells exhibit the ability of re-initiating the tumor. Therefore, the analysis of the complexity of the functional role of CSC in the context of neoplasia deserves further studies.

CSC show similar surface markers as normal stem cells in a given tissue, but a number of cell surface and intracellular biomarkers are commonly used to identify CSC among differentiated tumor cells and to isolate them. These markers include the clusters of differentiation (CD) CD44, CD24, or CD133, among others, the epithelial cell adhesion molecule (EpCAM) or the intracellular markers aldehyde dehydrogenase-1 (ALDH1) and the Notch, Wnt/β-catenin, Nanog, Sox2 pathways (for a more extensive review of CSC biomarkers in different tumors please see [20]). CD44 is expressed in CSC, as well as in a variety of normal stem cells, and plays an important role in CSC self-renewal and proliferation, leading to tumor growth, tumor metastasis, the activation of stemness transcription factors such as Nanog, Sox2, and Oct4, and chemotherapy resistance [21]. CD44 is a highly conserved surface glycoprotein encoded by the CD44 gene. The pre-mRNA contains 20 exons, where exons 1–5 and 16–20 are constant exons that lead to the standard form of 85 kDa while the remaining 10 exons (exons 6–15) are variant exons subjected to alternative splicing to produce the different CD44 variant forms [22]. The CD44v isoform is predominantly expressed in CSC over normal stem cells [23]. CD24 is a cell surface protein, heavily glycosylated, that plays an important role in cell-cell and cell-matrix interactions [24]. The expression of CD24 has commonly been investigated in combination with CD44 and other markers. For instance, high expression of CD44 and low expression of CD24 (CD44^+^/CD24^−/low^) together with expression of ALDH1 is a feature of breast cancer stem cells as compared to non-stem breast cancer cells [25]. Nevertheless, the expression of CD24 is variable among cancer cells [26]. ALDH1 has been reported as a CSC marker in adult tumors and, specially, is a bona fide marker of breast normal and cancer stem cells [27]. Interestingly, the ALDH1A3 isoform is predominantly expressed in CSC over normal stem cells [28]. The function of ALDH1 in CSC differentiation has been associated to its function in the oxidation of retinol to retinoic acid [29]; furthermore, positive ALDH1 expression has been reported to be correlated with chemotherapy resistance and poor prognosis [30].

**Figure 1:**
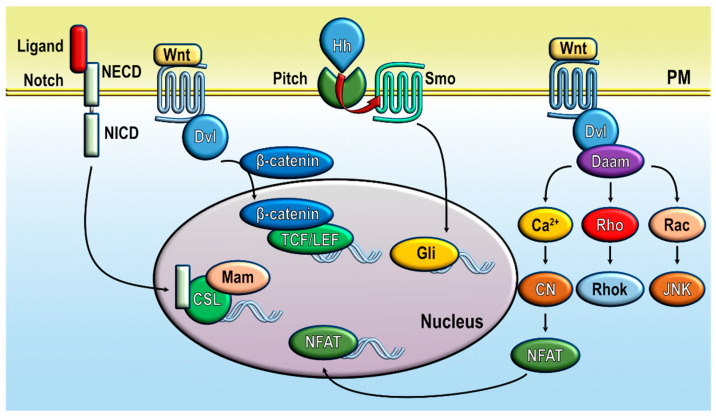
Notch, Wnt, and Hedgehog pathways in CSC. NECD, Notch extracellular domain; NICD, Notch intracellular domain; Mam, Mastermind; Dvl, Dishevelled; TCF/LEF, T-cell factor/lymphoid enhancer factor; Hh, hedgehog; Smo, Smoothened; Gli, glioma-associated oncogene; Daam, Dishevelled associated activator of morphogenesis; RhoK, Rho-kinase; CN, calcineurin; NFAT, nuclear factor of activated T-cells.

Different signaling pathways and transcription factors have been reported to play an essential role in the state of cell stemness. Among them, developmental signaling pathways such as Notch, Wnt/β-catenin or Hedgehog play important roles in normal stem cell function. Notch signaling pathway is activated by the interaction of ligands of the DSL family with the receptor protein Notch, a single-pass transmembrane protein. The interaction of the ligand with the Notch extracellular domain (NECD) leads to the cleavage and release of the Notch intracellular domain (NICD) that acts as a transcription factor and interacts with the transcription factors of the CSL family and Mastermind [31]. The CSL-Notch-Mastermind transcription factor complex up-regulates transcription of Notch-responsive genes leading to cell proliferation and promoting the formation of CSC colonies in different cancer types, including glioma and colon and breast cancer [32] (Figure 1).

The Hedgehog (Hh) pathway also plays a major role in normal stem cell and CSC biology and tumorigenesis. Hh signaling begins with the interaction of Hh ligands (Sonic Hedgehog, Indian Hedgehog, and Desert Hedgehog) with Patched-1, and to a lesser extent Patched-2, a twelve-pass transmembrane protein receptor. This process internalizes the Patched receptor and relieves the constitutive repression of the G-protein-coupled receptor Smoothened, which, in turn, leads to the nuclear translocation or the transcription factor Glioma-associated oncogene (Gli) resulting in the transcription of Hh target genes [33] (Figure 1). The Hh pathway has been associated to chemotherapy resistance and disease relapse [33].

The Wnt/β-catenin signaling pathway promotes CSC self-renewal while reducing the differentiation of CSC to proliferating tumor cells [32]. The Wnt ligands, a large family of secreted glycoproteins, interact with a Frizzled receptor in the plasma membrane, which signaling through the protein Dishevelled (Dvl) leads to inhibition of phosphorylation and proteasomal degradation of the protein β-catenin. Then, β-catenin accumulates in the cytosol and translocates into the nucleus, thus promoting the transcript of Wnt target genes by a mechanism involving the T-cell factor and lymphoid enhancer factor-1 (TCF/LEF1) transcription factors [34] (Figure 1). In addition to the activation of the β-catenin/TCF/LEF1 transcriptional pathway, Wnt proteins can induce alternative or non-canonical signaling pathways. In this pathway, Dvl is linked through Daam1 (Dishevelled associated activator of morphogenesis 1) to allow activation of the small GTPases Rho and Rac, which, in turn, activate Rho-kinase and JNK, respectively. Another non-canonical Wnt process is the Wnt/Ca^2+^ pathway. The interaction of Wnts with Frizzled, a family of G-protein coupled receptors, leads to the activation of phospholipase C and, thus, IP_3_ (inositol 1,4,5-triphosphate)-dependent Ca^2+^ release from the intracellular stores and subsequent Ca^2+^ influx across the plasma membrane [35]. The intracellular calcium fluxes induce the activation of downstream effects, such as PKC, CaMKII or calcineurin, thus leading to the nuclear translocation and activation of NFAT (nuclear factor of activated T-cells; Figure 1) [36]. A reciprocal interaction between Wnt signaling and NF-κB has been reported to play a key role in the progression of inflammation and cancer [37].

## 2. Calcium Signaling in Cancer Stem Cells and Cancer Hallmarks

Calcium ion modulates a myriad of physiological processes, such as muscle contraction, secretion or gene transcription, through a sophisticated and well-orchestrated machinery that deftly tunes cytosolic Ca^2+^ concentration [38]. In addition, Ca^2+^ participates in several pathological conditions, including cancer. Resistance to apoptosis and chemotherapy, high proliferation rate or the ability to migrate and to invade different tissues, have been considered key features in all cancer types for years [39]. As stated above, CSCs share some of these properties, but they also exhibit unique abilities such as capability for self-renewal [4,5]. All those processes are modulated by Ca^2+^ [40], thus, in recent years, aberrant expression of the proteins that control Ca^2+^ homeostasis has been included as a cancer hallmark.

Increases in intracellular Ca^2+^ concentration are required to trigger several Ca^2+^-dependent downstream effectors that modulate cellular pathways, such as calmodulin, which is essential for cell cycle and proliferation [41], NFAT proteins, with a role in cell cycle, differentiation or tumorigenesis [42], or the mitogen-activated protein kinase/extracellular signal-regulated (MAPK/ERK) pathway, involved in cancer cell survival, metastasis and chemotherapy resistance [43,44]. Increments of cytosolic Ca^2+^ concentration is achieved by the cells either by releasing Ca^2+^ from intracellular reservoirs, or by the opening of Ca^2+^-permeable channels in the plasma membrane (PM), which ensures an unlimited source of Ca^2+^ influx from the extracellular medium. Recent reports have demonstrated that CSC exhibit altered function in those mechanism (Table 1). Furthermore, it has been demonstrated that different signaling pathways that contribute to CSC pluripotency, such as the Wnt, TGF-β or FGF2, actively tune cytoplasmic Ca^2+^ concentration [45,46,47].

Concerning the Ca^2+^ release from intracellular stores, two calcium channels located in the endoplasmic reticulum (ER) membrane, IP_3_- and ryanodine (Ry)- receptors, are vital for CSC stemness, proliferation, and metastasis in different cancer types, such as glioblastoma [48], melanoma [49], and breast cancer [50].

Regarding Ca^2+^ entry, CSC present channels in the plasma membrane that are permeable to Ca^2+^ and could be gated by a variety of stimuli, such as voltage, second messengers or depletion of intracellular Ca^2+^ stores. For instance, over-expression of L- and T-type voltage-dependent Ca^2+^ channels is involved in tumorigenesis, proliferation, migration, and resistance to drugs in ovarian and glioblastoma CSC [51,52,53,54]. Moreover, abnormal expression of the voltage-dependent Ca^2+^ channel α2δ1 subunit, which modulates Ca^2+^ oscillation amplitude and the expression of different genes by keeping transcription factors in the nucleus, has started to be considered a tumoral marker in many cancers, such as lung [55,56], breast [57], and liver cancer [58] or laryngeal squamous [59], with a major role in CSC expansion. Different members of the transient receptor potential (TRP) channels, which are activated by several stimuli, such as temperature, pressure, and second messengers, participate in Ca^2+^ entry and have a key role in the CSC physiopathology of different cancers. For instance, TRPC3, which is over-expressed in triple-negative breast cancer cells, is activated by lysophosphatidic acid, promoting the process of self-renewal in CSC [60]. Enhanced expression or activation of TRPM7 has also been characterized in lung [61], glioblastoma [62], or neuroblastoma [63] and linked with several features of CSC. Similar findings have been found for TRPV2 channels-gated, among others, by the lipid ligand lysophosphatidylcholine [64] in the stemness of esophageal CSC [65]. Conversely, TRPV2 activation and expression promotes loss of stemness and apoptotic cell death in glioma [64,66,67] and hepatocellular carcinoma CSC [68]. This inverse correlation has also been observed for TRPA1 and TRPV1 channels in glioblastoma CSC [69].

Ca^2+^ reuptake, and the proteins involved, such as Ca^2+^-ATPases, Ca^2+^ exchangers or mitochondrial uniporter [70,71,72] might also play a role in CSC biology. A recent study has demonstrated that the sarco/endoplasmic Ca^2+^ ATPase (SERCA) presents an important antiapoptotic function in breast CSC, by reducing Ca^2+^-dependent apoptosis during glucose deprivation. This process is mediated by CaMK2α, which triggers the activation of NF-κB, and, in turn, SERCA over-expression [73]. Another report has shown that the mitochondrial Ca^2+^ uniporter and the Na^+^/Ca^2+^ exchanger, located in mitochondria and in the plasma membrane, respectively, are highly expressed in glioblastoma CSCs [74]; however, the relevance of such an aberrant protein expression remains yet unclear.

## 3. Store-Operated Calcium Entry in Cancer Stem Cells and Cancer Hallmarks

Store-Operated Calcium Entry (SOCE), a major mechanism for Ca^2+^ influx from the extracellular medium into excitable and non-excitable cells, is physiologically triggered by the activation of phospholipase C (PLC) and the production of IP_3_, which subsequently leads to the release of Ca^2+^ from intracellular stores, mainly the ER, resulting in the activation of store-operated calcium channels in the plasma membrane and a rapid increase in cytosolic Ca^2+^ concentration [75,76]. SOCE is an extremely complex biological mechanism, with high dependency on the pattern of expression of its components-STIMs, Orai, and TRPC proteins- and its modulators in each cell type. Since the last decades of the 20th century, several studies, both in vivo and in vitro, have reported that an altered expression pattern of the proteins that mediate SOCE leads to unbalanced Ca^2+^ homeostasis, which might contribute to tumor development, poor prognosis, and chemotherapeutic drug resistance [77].

The proteins of the STromal Interaction Molecule (STIM) family, STIM1 and STIM2, and their splice variants, possess a single transmembrane domain, with the N-region located either in the ER lumen or the extracellular medium, and a long cytosolic C-region [78,79]. Both, N- and C-terminal regions, present several key domains that enact STIM proteins’ double function upon a diminishment of the luminal Ca^2+^ concentration in the intracellular stores: (1) as the Ca^2+^ sensors of intracellular organelles, mediated by EF-hand Ca^2+^-binding domains in the N-terminus; and (2) as the transmitters of the filling state of intracellular Ca^2+^ stores to, and the activators of, Ca^2+^ channels in the plasma membrane. The latter is achieved by direct interaction between different domains within the STIM cytosolic C-region and the store-operated Ca^2+^ channels (STIM proteins structure is reviewed in [80,81,82]).

SOCE could be mediated by two types of channels with different biophysical properties: (1) the Ca^2+^ Release-Activated Ca^2+^ (CRAC) channels that exhibit high Ca^2+^ selectivity and an inwardly rectifying current, termed *I*_CRAC_, which its exclusively conducted by members of the Orai family [83]; and (2) the Store-Operated Ca^2+^ (SOC) channels, responsible to mediate a non-selective cation current denominated *I*_SOC_, formed by both, Orai1 and TRPC1, the first identified member of the canonical Transient Receptor Potential (TRPC) channel subfamily [84,85].

Orai1 was initially characterized as the main component of CRAC channel during a RNAi screening in 2006, when it was found that the Orai1 R91W mutation was responsible for abrogated CRAC channel function, critical for T-cell activation, in immunodeficient patients [86]. Orai1 and its paralogues, Orai2 and Orai3, present a unique structure among other Ca^2+^ channels, with four transmembrane domains spanning the PM and both, N- and C-terminus, facing the cytoplasm [87]. Originally, it was thought that Orai channels were formed by a homo-tetramer [88]; however, the crystal structure from *Drosophila melanogaster* Orai1 (dOrai1) presented a hexamer configuration, with the ion pore formed by the first transmembrane domain of the Orai subunits and located in the center of the complex surrounded by the remaining Orai plasma membrane domains [89]. The three members of the Orai family are capable to mediate store dependent Ca^2+^ influx, each of them with different biophysical properties that are extensively discussed here [90,91]. Some years ago, a shorter splicing variant for Orai1, Orai1β, lacking 64 aa in the N-terminus but able to generate functional Orai1 channels, was identified. Orai1β can be fully activated by STIM1 in a store-dependent manner but exhibits differential inactivation patterns as compared with the long variant, Orai1α [85]. In addition, recent studies have shown that Orai proteins might have a role in non-capacitative Ca^2+^ influx forming heteromers, such as the arachidonate-regulated Ca^2+^ channels (ARC), where three Orai1 and two Orai3 subunits form a pentamer [92], or interacting with other proteins to mediate store-independent Ca^2+^ influx [93].

TRPC1 belongs to the TRP channel superfamily, whose members ubiquitously mediate ion fluxes across the whole animal kingdom in a cell type-dependent manner [94]. All TRPs possess a similar structure with six transmembrane domains and the pore located between the 5th and 6th transmembrane regions. TRPs exhibit N- and C-terminus of variable length, containing the TRP box and different functional domains, subfamily-dependent, which participate in the functions of TRP channels and their relationship with other molecules and proteins. A functional TRP channel is composed by four TRP subunits forming either a homo- or hetero-tetramer [95,96]. Prior to Orai1 characterization, TRPC1 was a suggested candidate as the channel responsible for SOCE as STIM1 is able to interact and activate TRPC1 channels [97,98]. The current hypothesis suggests that TRPC1, together with Orai1, is involved in the generation of *I*_SOC_ currents [85,99,100,101]. TRPC1 channels, permeable to Na^+^, Ca^2+^, and Cs^+^ [102], are less selective for Ca^2+^ than Orai1 and allow a massive ion influx from the extracellular medium, required for the maintenance of SOCE and store replenishment [103].

**Figure 2:**
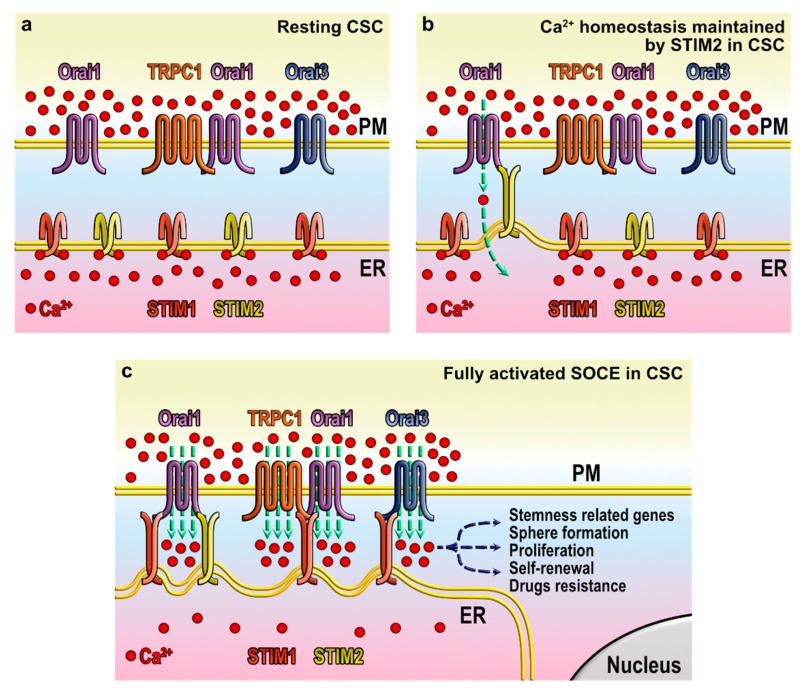
Store-operated Ca^2+^ in cancer stem cells. Cartoon depicting a hypothetical mechanism of SOCE in CSC, based on our current knowledge in non-tumoral cells: (**a**) when intracellular Ca^2+^ stores are filled, STIM proteins remain in their quiescent conformation; (**b**) small changes in luminal Ca^2+^ concentration are controlled by STIM2, briefly activating CRAC channels to replenish the reservoirs; (**c**) massive Ca^2+^ store depletion triggers the activation of STIM proteins and the channels in plasma membrane, resulting in the critical increase in cytosolic Ca^2+^ required for tumorigenic responses.

Several stimuli might trigger intracellular Ca^2+^ stores depletion that will be sensed by STIM proteins (Figure 2). Minor reductions in luminal Ca^2+^ concentration will be detected by STIM2, which in turn, would momentarily trigger the opening of CRAC channels, allowing Ca^2+^ influx from the extracellular medium that will quickly be reintroduced into the stores by Ca^2+^-ATPase pumps to revert to resting conditions (Figure 2b) [80]. More extensive discharge of intracellular Ca^2+^ stores would trigger the activation of STIM1, in addition to STIM2, which will fully generate the opening of CRAC channels, subsequently followed by a rapid and transient Ca^2+^ entry [83,104,105,106]. Ca^2+^ entry conducted by Orai1 will be severely inhibited after few milliseconds by Ca^2+^ itself [107,108] as well as after a longer period of time by the interaction of Orai1 N- and C-terminus with different proteins, such as SARAF [109,110,111] or by Orai1 serine phosphorylation at the N-terminus by kinases such as PKC or PKA [112,113]. Ca^2+^ influx through Orai1 leads to the recruitment of TRPC1 at the plasma membrane, which conducts further Ca^2+^ influx to reach the critical cytosolic Ca^2+^ concentration required for the physiological response evoked by the stimulus (Figure 2c) [103,114]. Next, the excess of intracellular Ca^2+^ is speedily removed, either by reintroducing the ion into the ER or by its extrusion to the extracellular medium via Ca^2+^-ATPases [70,71]. When agonist stimulation ceases, replenishment of the Ca^2+^ stores leads to the incorporation of Ca^2+^ to STIM1/2 EF-hand domains, which return these proteins to their quiescent conformation, leading to the deactivation of SOCE [79,104].

The number of studies linking SOCE proteins with cancer stem cell properties is growing at an amazingly fast pace; however, our knowledge is still extremely limited. Regarding STIM proteins, it is known that STIM1 associates with the hypoxia-inducible factor-1 alpha (HIF-1α) modulating each other, in a reciprocal dependency, in hypoxic hepatocarcinoma cells (HCCs). HIF-1α up-regulates STIM1 transcription, which in turn, induces higher SOCE, activating the CaMKII and P300 pathways, which are required for the accumulation of HIF-1α in HCCs [115].

Even less is known about the role of TRPC1 in CSC, since some of the inhibitors used to block SOCE, act over both Orai1 and TRPC1 channels. For instance, treatment with SKF96365, a SOCE inhibitor, impairs CSC proliferation in the glioblastoma stem-like cell line, TG1, triggering these cells to adopt a quiescent state by up-regulation of *CDKN1A* and *G0S2* and the down-regulation of *CCNB1* genes [116]. Similarly, SOCE impairment by SKF96365 in liver cancer stem cells (LCSCs) resulted in a drastic reduction in their ability to form spheroids, suppressing at the same time the expression of stemness-related genes. SOCE is activated in LCSC via the fibroblast growth factor 19 (FGF19), promoting the nuclear translocation of NFATc2 and self-renewal [117]. Even when the expression of Orai and STIM proteins was checked in both studies, TRPC1 was not considered and might be a possible candidate for future approaches.

## 4. Functional Role of Orai in Cancer Stem Cells and Cancer Hallmarks

As described above, native CRAC channels are hexameric structures comprised by the heteromeric association of Orai1, Orai2, and Orai3. Although all Orai family members can conform the channel, Orai2 and Orai3 also act as Ca^2+^ current modulators due to their lower Ca^2+^ conductivity and greater fast Ca^2+^-dependent inactivation as compared to Orai1 [108,118]. Several studies have demonstrated that the three Orai proteins are overexpressed in tumor samples and different human cancer cell lines compared with their non-tumorigenic counterpart cell lines. Hence, Orai1 is overexpressed in oral/oropharyngeal squamous cell carcinoma cells (OSCC) [119,120], liver [121], and breast cancer cells [122,123], Orai2 expression is increased in gastric [124], breast [125], oral [120], and acute myeloid leukemia cancer cells [126], while Orai3 expression is enhanced in the luminal breast cancer subtype [123,127], as well as in lung [128], pancreatic [129], and prostate cancer cells [130] (for a more extensive review see [131,132,133,134,135]). Using pharmacological or gene silencing approaches, to inhibit protein function or to avoid protein expression, respectively, the mentioned studies showed that Orai proteins play a crucial role in both tumorigenesis and the development and maintenance of different cancer hallmarks, including resistance to apoptosis, proliferation, migration, invasion, and metastasis via SOCE. However, as mentioned above, Orai1 can also mediate cancer progression by regulating and driving different Ca^2+^ influx pathways that are independent of the filling state of intracellular Ca^2+^ stores [93]. These pathways include: (1) the arachidonic acid-regulated Ca^2+^ current mediated by a Orai1/3 channel [130,136,137], (2) the constitutive Ca^2+^ influx mediated by the physical interaction between Orai1 and secretory pathway Ca^2+^-ATPase-2 [138,139,140], and (3) the Ca^2+^ influx mediated by the physical and functional interaction of Orai1 with the small conductance Ca^2+^-activated K^+^ channel 3 [141,142] or with the voltage-dependent Kv10.1 channel in the plasma membrane [143,144]. In the latter, a reciprocal positive feedback loop promotes the activation of both K^+^ channels by Orai1-mediated Ca^2+^ entry, which in turn leads to plasma membrane hyperpolarization, thus maintaining the driving force for Ca^2+^ influx and Ca^2+^ entry through Orai1 channels [141,145,146].

The role of Orai family proteins has also been described in the induction of CSC phenotype in a variety of cancers, such as glioblastoma, lung, and OSCC cancer cells. This CSC phenotype includes self-renewal capacity, tumor spheres formation, drug resistance, increased migration ability, and enhanced expression of stemness-related transcription factors and CSC-related markers [119,147,148]. Lee et al. demonstrated that Orai1, the predominant Orai family member in OSCC, is overexpressed in OSCC-derived CSC and its function is required for the maintenance of stemness and CSC phenotype through NFAT signaling pathway. Hence, Orai1 mediates the enhanced expression of stemness-related transcription factors, such as Nanog, Oct4 or Sox2, and promotes some CSC-related markers, including an increased ALDH1 activity and a higher CSC-related gene expression (Ezh2, Gli1, Hes1, Zeb2, FGF4, and IL4). The inhibition of Orai1 function in human tongue squamous carcinoma cell lines SCC4 and HOK-16B BapT by a pharmacological approach, using the Orai1 specific small molecular blocker compound 5D, impaired self-renewal capacity and reduced migration and invasion abilities in these cancer cells. Comparable results were also obtained by two different genetic approaches, using a specific siRNA to reduce Orai1 gene expression and inducing the overexpression of an Orai1 dominant negative mutant. Furthermore, Orai1 overexpression using viral vectors promoted CSC phenotype in non-tumorigenic immortalized oral epithelial cells HOK-16B [119]. Using related approaches, Singh et al. demonstrated that Orai1 and Orai2 overexpression is required for cell proliferation, migration, and colonization in SAS human tongue carcinoma cell line, processes that were found to be dependent on Akt/mTOR/NF-κB signaling pathway activation [120]. Analogous results were reported in glioblastoma stem cells derived from different human glioblastoma surgical samples. In these cells, the treatment with YM-58483, a CRAC current inhibitor, or with GSK-7975A, a more specific inhibitor of Orai1-mediated Ca^2+^ current, promoted a decrease in Sox2 expression, effect that was associated with reduced spheres formation and with the inhibition of their proliferation and self-renewal capacities [148]. Orai1 has been also related with chemoresistance, event that has been widely associated with CSC phenotype in cancer cells as previously mentioned. Hence, it has been demonstrated that ectopic overexpression of Orai1, using a plasmid vector, inhibited 5-fluorouracil-induced cell death in HepG2 hepatocarcinoma cells; meanwhile, Orai1 gene expression knockdown promoted the autophagic cell death induced by this pharmacological compound [121]. Similar findings were observed in cisplatin-resistant A2780 ovary carcinoma cells, in which Orai1 expression and SOCE are increased compared to therapy-sensitive parental cells. Pharmacological inhibition of Orai1 in cisplatin-resistant A2780 cells, using 2-aminoethoxydiphenyl borate (2-APB), promoted cisplatin-induced apoptotic cell death similarly to those observed in therapy-sensitive A2780 cells [149]. Conversely, an opposite effect has been reported in prostate cancer cells since the downregulation of Orai1 expression, caused by steroid-deprived conditions or by using specific siRNA against Orai1, and the impairment of Orai1 function by the overexpression of two Orai1 mutants, Orai1 R91W and Orai1 L273S, prevented the apoptotic cell death induced by different pharmacological compounds, including thapsigargin, TNFα, cisplatin, and oxaliplatin. Furthermore, the restoration of Orai1 expression in steroid-deprived cells by transfection with a Orai1 plasmid vector promoted the loss of chemoresistance in these cells [150].

Regarding the role of Orai3 in the CSC phenotype acquisition in cancer cells, it has been demonstrated that Orai3 overexpression is correlated with tumoral aggressiveness and chemoresistance acquisition in breast cancer cells [127,147]. Orai3 stable overexpressing T47D and MCF7 clones exhibited resistance to apoptotic cell death induced by thapsigargin, cisplatin, 5-fluorouracil, and paclitaxel compared with their parental cells transfected with the empty vector. This Orai3-dependent chemoresistance is acquired by ubiquitin ligase Nedd4-2-mediated p53 ubiquitination via the PI3K/Sgk-1 signaling pathway [127]. Previously, the same group demonstrated that Orai3 expression is also positively correlated with the oncogene c-myc expression in the ER-positive (luminal-like) breast cancer cell line MCF7 [151]. Daya et al. revealed that chemotherapy treatment increased Orai3 expression in primary human lung adenocarcinoma cells derived from bronchial biopsy specimens. Similar findings were reported in lung adenocarcinoma cell lines A549 and NCI-H23 after treatment with cisplatin. Interestingly, cisplatin treatment increased SOCE without affecting the expression of other proteins involved in CRAC current activation, such as STIM1, STIM2, and Orai1, even a slight decrease in the expression of Orai1 was observed in A549 cells. Orai3 gene expression knockdown using a specific siRNA enhanced cisplatin-induced apoptotic cell death in both lung adenocarcinoma cell lines, while Orai3 overexpression drastically reduced cisplatin-induced cell death and enhanced stemness in non-small cell lung cancer cells, as demonstrated by the enhanced expression of the stemness-related transcription factors Nanog and Sox2 via PI3K/AKT pathway, which resulted to be dependent on the increase in Orai3 expression [147].

## 5. Conclusions

Altogether, the presented data support an essential role of SOCE mediators-STIMs, Orais, and TRPC proteins in the induction of CSC phenotype. However, our current understanding about the role of these proteins in cancer stemness is incomplete since the existing studies do not take in consideration their participation in other pathways, such as the store independent function of STIM1, Orai1, and Orai3, while the possible implications of Orai2 or TRPC1 in the stemness properties of CSC remain unclear.

## Figures and Tables

**Table 1:** Implications of calcium-related proteins in cancer stem cells. ND: not determined. VGCC: voltage-gated calcium channels.

Ca^2+^ Pumps and Exchangers					
Protein	Expression/Functional Change in CSC	CSC Type	Role in CSC	Signaling Pathway Activated	Ref.
SERCA	Overexpression	Breast cancer stem cells	Cell survival in glucose-deprived conditions	Decrease [Ca^2+^]_c_ and avoid Ca^2+^-dependent apoptosis during glucose deprivation	[73]
**ER Ca^2+^ channels**					
**Protein**	**Expression/Functional Change in CSC**	**CSC Type**	**Role in CSC**	**Signaling Pathway Activated**	**Ref.**
RyR1	HIF-depended activation	Breast cancer stem cells	Chemoresistance	PYK2/SRC/STAT3 signaling pathway	[50]
IP_3_R	Channel activation	Melanoma stem cells	Stemness maintenance	ND	[49]
	Channel activation	Glioblastoma stem cell	Cell self-renewal Chemoresistance	ND	[48]
**Non-SOCE channels**					
**Protein**	**Expression/Functional Change in CSC**	**CSC Type**	**Role in CSC**	**Signaling Pathway Activated**	**Ref.**
**VGCC**					
L- and T-type	Overexpression	Ovarian cancer stem cells	Tumor spheres formation Apoptosis resistance Stemness maintenance	Increase the transcription of Oct, Nanog, and Sox2 via ERK1/2 and AKT signaling pathways	[53]
T-type calcium channel	Overexpression (Cav3.2)	Glioblastoma stem cells	Apoptosis resistance Chemoresistance Stemness maintenance	Increase cell survival via AKT/mTOR pathways	[52]
	Overexpression	Glioblastoma stem cells	Apoptosis resistance	Stimulate Na^+^-dependent nutrient transport	[54]
α2δ1 subunit	Overexpression	Small cell lung cancer stem cells	Chemoresistance	MEK/ERK signal pathway??	[55]
α2δ1 subunit	Overexpression	Non-small cell lung cancer stem cells	Chemoresistance Cell Survival Stemness maintenance	Notch3 activation via Ca^2+-^Calcineurin/NFATc2 signaling pathway	[56]
	Overexpression	Breast cancer stem cells	Stemness maintenance Cell self-renewal	ND	[57]
	Overexpression	Hepatocellular cancer stem cells	Cell self-renewal Cell survival Stemness maintenance	ERK1/2 MAPK signaling pathway	[58]
	Overexpression	Laryngeal squamous cancer stem cells	Tumor spheres formation Chemoresistance Tumorigenesis Stemness maintenance	ND	[59]
**Protein**	**Expression/Functional Change in CSC**	**CSC Type**	**Role in CSC**	**Signaling Pathway Activated**	**Ref.**
**TRP Channels**					
TRPC3	Overexpression	Breast cancer stem cells	Cell self-renewal	Increase IL-8 secretion via LPA/LPAR3/TRPC3 pathway	[60]
TRPM7	Overexpression	Lung cancer stem cells	Tumor spheres formation Stemness maintenance	Hsp90α/uPA/MMP2 signaling pathway	[61]
	Channel activation	Glioblastoma stem cells	Stemness maintenance Cell proliferation, migration and invasion	STAT3 and Notch signaling pathways	[62]
	Overexpression	Neuroblastoma stem cells	Stemness maintenance	ND	[63]
TRPA1	Channel activation Overexpression	Glioma stem cells	Cell differentiation Apoptotic cell death Stemness loss	ND	[69]
TRPV1	Channel activation Overexpression	Glioma stem cells	Cell differentiation Apoptotic cell death Stemness loss	ND	[69]
TRPV2	Overexpression	Esophageal squamous cancer stem cells	Stemness maintenance Cell proliferation	ND	[65]
	Channel activation Overexpression	Glioblastoma stem cells	Stem cell differentiation Reduce self-renewal capacity Apoptotic cell death	AKT-PI3K/RPS6KBI/PTEN signaling pathway	[64,66,67]
	Channel activation Overexpression	Liver cancer stem cells	Impair tumor spheres formation and self-renewal capacity Stemness loss	ND	[68]
**SOCE channels**					
**Protein**	**Expression/Functional Change in CSC**	**CSC Type**	**Role in CSC**	**Signaling Pathway Activated**	**Ref.**
Orai1	Overexpression	oral/oropharyngeal squamous cancer stem cells	Tumor spheres formation Cell self-renewal Stemness maintenance	NFAT signaling pathway	[119]
	Overexpression	Glioblastoma stem cell	Tumor spheres formation Cell self-renewal Stemness maintenance	ND	[148]
Orai3	Overexpression	Non-small cell lung cancer stem cells	Chemoresistance Stemness maintenance	PI3K/AKT signaling pathway	[147]
SOC Channels	Channel activation	Glioblastoma stem cell	Cell proliferation	Up-regulation of *CDKN1A* and *G0S2* and the down-regulation of *CCNB1* genes	[116]
	Channel activation	Liver cancer stem cells	Stemness maintenance Tumor spheres formation Cell self-renewal	FGF19/SOCE/NFATc2 signaling pathway	[117]

## Data Availability

The data presented in this study are available on request from the corresponding author.
